# Team-based continuity of care for patients with hypertension: a retrospective primary care cohort study in Hong Kong

**DOI:** 10.3399/BJGP.2023.0150

**Published:** 2023-10-17

**Authors:** Wanchun Xu, Esther Yee Tak Yu, Weng Yee Chin, Ivy Lynn Mak, Cheyenne I Ying Chan, Cindy Lo Kuen Lam, Eric Yuk Fai Wan

**Affiliations:** Department of Family Medicine and Primary Care, Li Ka Shing Faculty of Medicine, University of Hong Kong, Hong Kong Special Administrative Region (SAR).; Department of Family Medicine and Primary Care, Li Ka Shing Faculty of Medicine, University of Hong Kong, Hong Kong Special Administrative Region (SAR).; Department of Family Medicine and Primary Care, Li Ka Shing Faculty of Medicine, University of Hong Kong, Hong Kong Special Administrative Region (SAR).; Department of Family Medicine and Primary Care, Li Ka Shing Faculty of Medicine, University of Hong Kong, Hong Kong Special Administrative Region (SAR).; Department of Family Medicine and Primary Care, Li Ka Shing Faculty of Medicine, University of Hong Kong, Hong Kong Special Administrative Region (SAR).; Department of Family Medicine and Primary Care, Li Ka Shing Faculty of Medicine, University of Hong Kong, Hong Kong SAR; Department of Family Medicine, University of Hong Kong, Shenzhen Hospital, Shenzhen.; Department of Family Medicine and Primary Care, and Department of Pharmacology and Pharmacy, Li Ka Shing Faculty of Medicine, University of Hong Kong, Hong Kong SAR.

**Keywords:** cardiovascular diseases, continuity of patient care, hypertension, primary health care

## Abstract

**Background:**

Continuity of care (COC) is associated with improved health outcomes in patients with hypertension. Team-based COC allows more flexibility in service delivery but there is a lack of research on its effectiveness for patients with hypertension.

**Aim:**

To investigate the effectiveness of team-based COC on the prevention of cardiovascular disease (CVD) and mortality in patients with hypertension.

**Design and setting:**

A retrospective cohort study in a primary care setting in Hong Kong.

**Method:**

Eligible patients included those visiting public primary care clinics in Hong Kong from 2008 to 2018. The usual provider continuity index (UPCI) was used to measure the COC provided by the most visited physician team. Cox regression and restricted cubic splines were applied to model the association between the COC and the risk for CVDs and all-cause mortality.

**Results:**

This study included 421 640 eligible patients. Compared with participants in the lowest quartile of UPCI, the hazard ratios for overall CVD were 0.94 (95% CI = 0.92 to 0.96), 0.91(95% CI = 0.89 to 0.93), and 0.90 (95% CI = 0.88 to 0.92) in the second, third, and fourth quartiles, respectively. A greater effect size on CVD risk reduction was observed among the patients with unsatisfactory blood pressure control, patients aged <65 years, and those with a Charlson comorbidity index of <4 at baseline (*P*interaction<0.05 in these subgroup analyses), but the effect was insignificant among the participants with an estimated glomerular filtration rate of <60 ml/ min/1.73 m^2^ at baseline.

**Conclusion:**

Team-based COC via a coordinated physician team was associated with reduced risks of CVD and all-cause mortality among patients with hypertension, especially for the patients with unsatisfactory blood pressure control. Early initiation of team-based COC may also achieve extra benefits.

## INTRODUCTION

Hypertension is a highly prevalent medical condition worldwide,^1^ which is also one of the most critical risk factors of cardiovascular disease (CVD) and premature death.^2^ The monitoring and care of patients with hypertension relies mainly on the primary care system, where continuity of care (COC) is considered a crucial component of primary care practice.^3^ Continuity of care refers to the relational, informational, and managerial continuity in the provision of care for patients. Traditional COC in primary care is mainly viewed as the relationship between the patient and a designated practitioner that extends beyond specific episodes of illness or diseases,^4^ which could also be termed individual-based COC. Continuity of care was found to increase the chance of achieving blood pressure targets and improved quality of life^5^^–^7 in patients with hypertension. However, assigning a physician to the patient for long-term COC requires that the primary care system be adequately and appropriately staffed, which is challenging in many public healthcare sectors, especially for those in resource- restricted settings.^8^

In contending with the tension mentioned above, achieving COC on the team-based level has gained attention in primary care, as the physician group practice is becoming popular in the current climate around the world.^9^^,^^10^ The physician group practice has been shown to improve the quality and efficiency of care because the previous one-to-one physician–patient relationship is leveraged to connect the patient to a team of physicians.^11^^,^^12^ With more flexibility in scheduling appointments for physician encounters, team-based COC via a coordinated physician team can also be a feasible alternative to provide longitudinal continuous care for patients.^11^ Such an approach places greater emphasis on managerial and informational continuity, where shared management plans, care protocol, and information sharing within the physician team can serve as complementary methods to achieve COC.^4^^,^^13^ Team-based COC via a physician team has been found to effectively prevent CVD incidence and mortality among patients with diabetes.^14^ However, the effectiveness of team-based COC for patients with hypertension remains unknown. Therefore, this study aimed to investigate the effect of team-based COC on the risk of CVDs and mortality among patients with hypertension.

**Table:** How this fits in

Continuity of care (COC) is associated with improved health outcomes in patients with hypertension. Team-based COC can transform the previous one-to-one, physician–patient relationship by connecting the patient to a coordinated group of physicians, thereby helping to sustain COC for patients with hypertension in the long-term follow-up. This study showed clinically significant effects of team-based COC in reducing the risks of CVD incidence and mortality among patients with hypertension. Team-based COC via a physician group can serve as a feasible and effective approach for achieving COC in primary healthcare systems with limited resources.

## METHOD

### Study design and participants

This is a retrospective cohort study conducted in patients with hypertension in Hong Kong Special Administrative Area (SAR), China, where physician team practice has been implemented in some pilot public primary care clinics over the past decade.^14^ There are around three physicians in one typical physician team at one time, and each physician is posted to a clinic for 2 to 5 years, during which time they work in a fixed physician team. Patients are allowed to book appointments for office visits across different physicians within the same team.^14^ In the event of a temporary absence of a physician, the patients are advised to reschedule their appointments or to make appointments with another physician in the same team. Locum doctors would fill in temporarily during the longer absence of physicians to ensure the operation of team- based COC. In this study electronic medical records were extracted from the clinical management system (CMS) of the Hong Kong Hospital Authority (HA). The CMS is the centralised electronic medical databases of public health sectors since 1994 and has been validated with high coding accuracy in cardiovascular diseases.^15^

**Figure 1.**
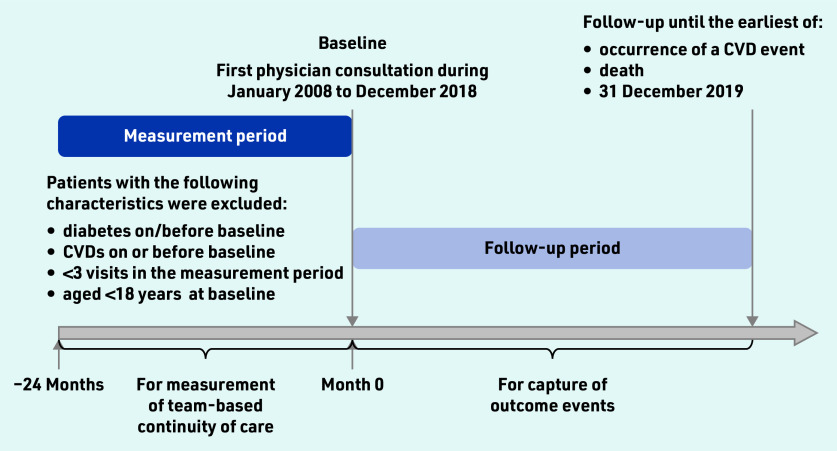
*Flowchart of study design. Team-based usual provider continuity index (UPCI) was calculated by dividing the number of attendances at the most visited physician team by the total number of attendances within the 2-year measurement period. Patients with <3 visits within the measurement period were excluded from the analysis to assure the accurate measurement of the UPCI. CVD = cardiovascular disease.*

Eligible participants were adult patients with hypertension who had at least one attendance at a public general outpatient clinic between January 2008 and December 2018. The earliest attendance date from 2008 to 2018 was set as the baseline date for each patient. A 2-year period before the baseline was defined as the measurement period for the degree of team-based COC, where the patients with fewer than three visits were excluded from the analysis to avoid the misclassification of COC degree due to a small number of visits. Patients with diabetes or cardiovascular diseases were also excluded from the analysis. Further details of the study design are illustrated in Figure 1. The list of case definitions based on International Classification of Diseases Clinical Modification (ICD)-9-CM/International Classification of Primary Care (ICPC)-2 codes is detailed in Supplementary Table S1. Each participant was followed till the occurrence of outcomes of interest, death, or 31 December 2019, whichever was earlier.

### Measurement of exposure, outcomes, and covariates

The usual provider continuity index (UPCI) is a common measure of continuity of care,^16^ which was modified to measure team- based COC. The modified UPCI to measure team-based COC is calculated by dividing the number of visits to the patient’s most visited physician team by the total number of physician consultations they attended within the measurement period (2 years before the baseline date). The UPCI was selected as the measurement for the primary analysis owing to its simplicity in interpretation, as it represents the proportion of a patient’s visits to the most frequently visited physician team. The team-based UPCI ranges from 0 to 1, with 0.5 indicating that the patient visited the same team for 5 out of every 10 attendances. The primary outcome was the risks of CVD events over the follow-up period (that is, any of coronary heart disease [CHD], stroke, and heart failure). The risks of CVD subtypes mentioned above and all-cause mortality were examined as secondary outcomes. Information on mortality was collected from the Hong Kong Death Registry. Details on baseline covariates were collected from the CMS database.

### Statistical analysis

Participants were classified into four groups based on the UPCI quartiles:

- <0.50;
- 0.50–0.70;
- 0.71–0.91; and
- 0.92–1.00.

This was to compare the risk of complications and mortality among varying degrees of COC. To minimise selection bias among groups, baseline characteristics were adjusted using fine stratified weights,^17^ in which propensity scores were generated with a multivariable logistic regression between the patient groups and baseline characteristics. A standardised mean difference (SMD) <0.1 indicates a good balance across groups.^18^ The hazard ratios (HR) for groups with higher UCPI relative to the group with UPCI <0.50 (the reference group) were examined using a multivariable Cox proportional hazard model, with adjustments for baseline characteristics, including: age, sex, smoking status, fasting glucose, blood pressure, lipid profile, body mass index, estimated glomerular filtration rate (eGFR), Charlson comorbidity index (CCI), the total number of attendances within 2 years before the baseline, and the usage of antihypertensive medications and lipid-lowering agents. Additionally, to test the linearity of the exposure–response relationship, the restricted cubic splines (3 knots) were applied to flexibly model the association between the COC and the outcomes of interest.^19^ Multiple imputation was applied to handle missing values at baseline (the data completion rate for the covariates were presented in Supplementary Table S2). Five sets of imputations were used and results were combined based on Rubin’s rules.^20^

Subgroup analyses were performed based on the patient’s sex, age (<65 years versus ≥65 years), smoking status (current smoker versus non-smoker), CCI (<4 versus ≥4), fasting glucose (≤5.6 versus >5.6 mmol/L), blood pressure (systolic blood pressure [SBP] <140 and diastolic blood pressure [DBP] <90 mmHg versus SBP ≥140 or DBP ≥90 mmHg), BMI (<23 versus ≥23 kg/m^2^), and eGFR (≥60 versus <60 ml/ min/1.73m^2^). Several sensitivity analyses on CVD risk were conducted, including an analysis without weighting, an analysis with complete cases, and an analysis that included only patients with at least 3 years of follow-up. Additional sensitivity analyses were conducted by dividing the patients into quartiles defined by the alternative measurements of continuity of care to test if the approach of continuity measurement would influence the results, including the Continuity of Care Index (COCI),^21^ the Modified Continuity Index (MMCI),^22^ and the Sequential Continuity Index (SECON)^23^ (Supplementary Table S3). To affirm that the inclusion criteria of at least three attendances had a negligible impact on the results, the associations were examined again by including patients with at least five attendances and at least eight attendances. To test if different prescribing patterns would influence the results, the authors conducted a sensitivity analysis additionally adjusted for the four prevalent types of antihypertensives (angiotensin-converting enzyme inhibitor and angiotensin receptor blockers [ACEI/ARBs], β-blockers, calcium channel blockers, and diuretics) and two commonly prescribed lipid-lowering agents (statin and fibrate) in the primary care settings.

All analyses were performed using Stata 16.1. A two-tailed *P*<0.05 was defined as statistically significant.

## RESULTS

### Patient characteristics

A total of 421 640 eligible patients were identified from the CMS database. Descriptive statistics of the baseline characteristics before weighting are displayed in Supplementary Table S4. The mean age of patients was 64.2 years, and 43.4% (*n* = 182 849) were males. The prescribing patterns regarding the antihypertensives and lipid-lowering agents were similar across different UPCI quartiles among the eligible patients. After multiple imputation and weighting, 95 patients were excluded owing to the lack of an available match. Baseline characteristics of patients after weighting are presented in Table 1. All baseline characteristics had a SMD of <0.1, indicating an adequate balance between groups.

**Table 1.** Baseline characteristics by continuity of care quartiles after multiple imputation and weighting

**Characteristic**	**Total (*N* = 421 545)**	**Quartile**				**SMD^a^**
		**UPCI <0.50 (*N* = 100 915)**	**UPCI 0.50–0.70 (*N* = 107 971)**	**UPCI 0.71–0.91 (*N* = 103 487)**	**UPCI 0.92–1.00 (*N* = 109 172)**	
Sex, male, *n* (%)	183 050 (43.4)	43 966 (43.6)	47 071 (43.6)	44 917 (43.4)	47 097 (43.1)	<0.01
Age, years, mean (SD)	64.0 (12.2)	63.8 (12.1)	63.9 (12.3)	64.2 (12.0)	64.2 (12.2)	0.03
Smoker, *n* (%)	22 692 (5.4)	5434 (5.4)	5602 (5.2)	5592 (5.4)	6064 (5.6)	0.02
SBP, mmHg, mean (SD)	134.6 (15.8)	134.7 (16.1)	134.4 (15.9)	134.6 (15.5)	134.7 (15.5)	0.02
DBP, mmHg, mean (SD)	76.3 (10.6)	76.5 (10.7)	76.3 (10.7)	76.3 (10.4)	76.3 (10.5)	0.02
Fasting glucose, mmol/L, mean (SD)	5.3 (0.6)	5.3 (0.6)	5.3 (0.6)	5.3 (0.6)	5.3 (0.5)	<0.01
BMI, kg/m^2^, mean (SD)	25.2 (3.8)	25.2 (3.8)	25.2 (3.9)	25.1 (3.8)	25.1 (3.9)	<0.01
LDL-C, mmol/L, mean (SD)	3.2 (0.8)	3.2 (0.8)	3.2 (0.8)	3.2 (0.8)	3.2 (0.8)	<0.01
eGFR, ml/min/1.73m^2^, mean (SD)	102.5 (63.3)	102.8 (29.4)	102.4 (29.6)	102.4 (72.8)	102.5 (93.8)	0.01
CCI, mean (SD)	3.4 (2.8)	3.4 (2.8)	3.4 (2.8)	3.4 (2.8)	3.4 (2.8)	0.02
Use of anti-hypertensive drugs, *n* (%)	401 385 (95.2)	96 485 (95.6)	103 371 (95.7)	98 440 (95.1)	103 088 (94.4)	0.06
Use of lipid-lowering agents, *n* (%)	40 622 (9.6)	9741 (9.7)	10 459 (9.7)	9977 (9.6)	10 445 (9.6)	<0.01
Number of appointments, mean (SD)	11.7 (4.9)	11.7 (5.5)	11.7 (4.9)	11.8 (4.4)	11.7 (4.6)	0.02
UPCI, mean (SD)	0.69 (0.24)	0.36 (0.08)	0.58 (0.06)	0.80 (0.06)	0.98 (0.03)	NA

a *Standardised mean difference (SMD) listed is the largest SMD between any pairs of the groups. BMI = body mass index. CCI = Charlson comorbidity index. DBP = diastolic blood pressure. eGFR = estimated glomerular filtration rate. LDL-C = low-density lipoprotein — cholesterol. NA = not applicable. SBP = systolic blood pressure. SD = standard deviation. UPCI = usual provider of care index.*

### Association between UPCI and outcomes of interest

**Figure 2.**
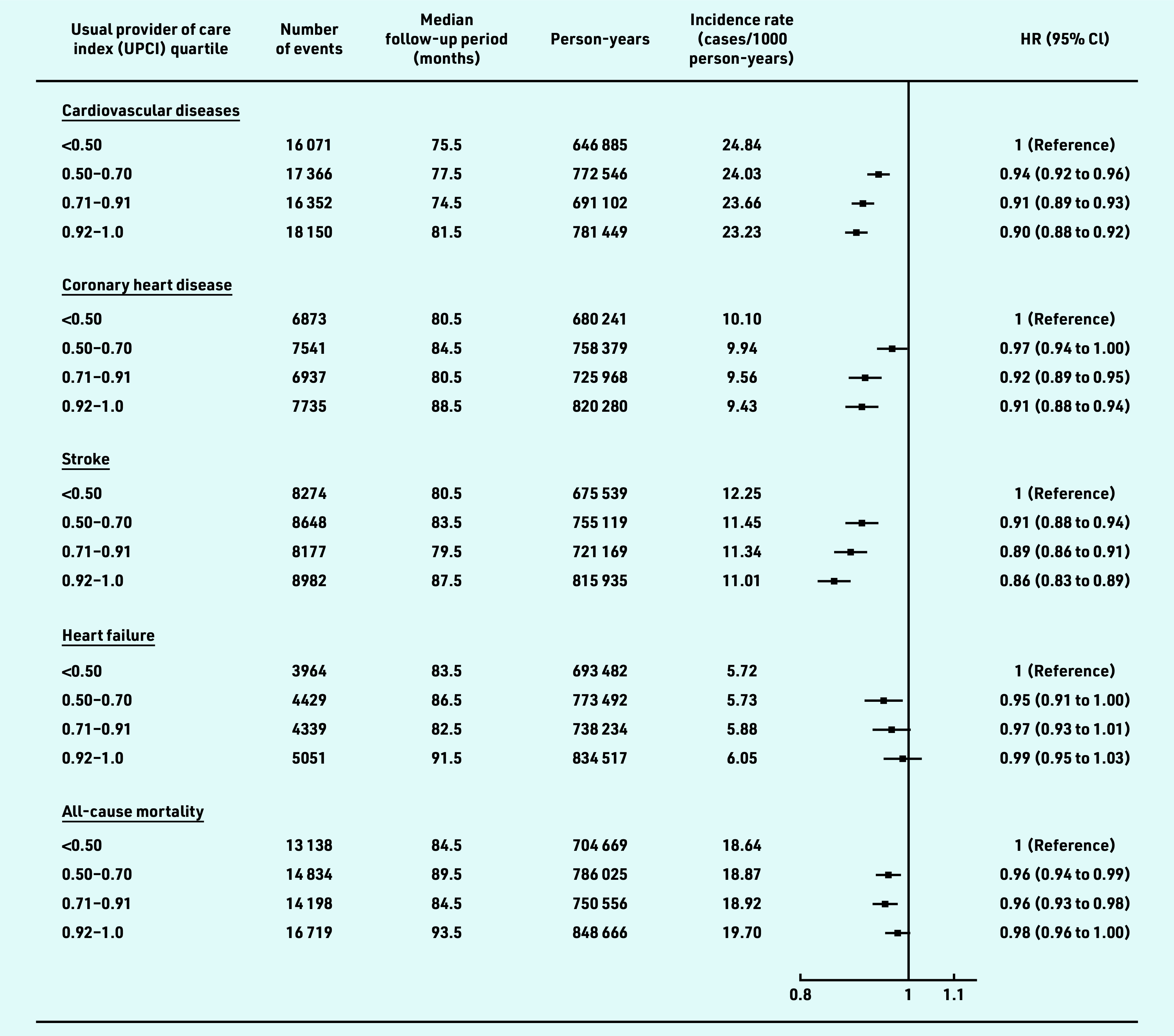
*Association of team-based continuity of care with risks of CVD and all-cause mortality among patients with hypertension. Participants were classified into four groups based on UPCI quartiles. Hazard ratios were adjusted for age, sex, smoking status, systolic and diastolic blood pressure, body mass index, fasting glucose, low-density lipoprotein cholesterol, estimated glomerular filtration rate, Charlson comorbidity index, number of medical attendances, use of antihypertensive drugs, use of lipid-lowering drugs, and use of antidiabetic drugs at baseline. CVD includes coronary heart disease, heart failure, and stroke. CVD = cardiovascular disease. HR = hazards ratio. UPCI = usual provider of care index.*

**Figure 3.**
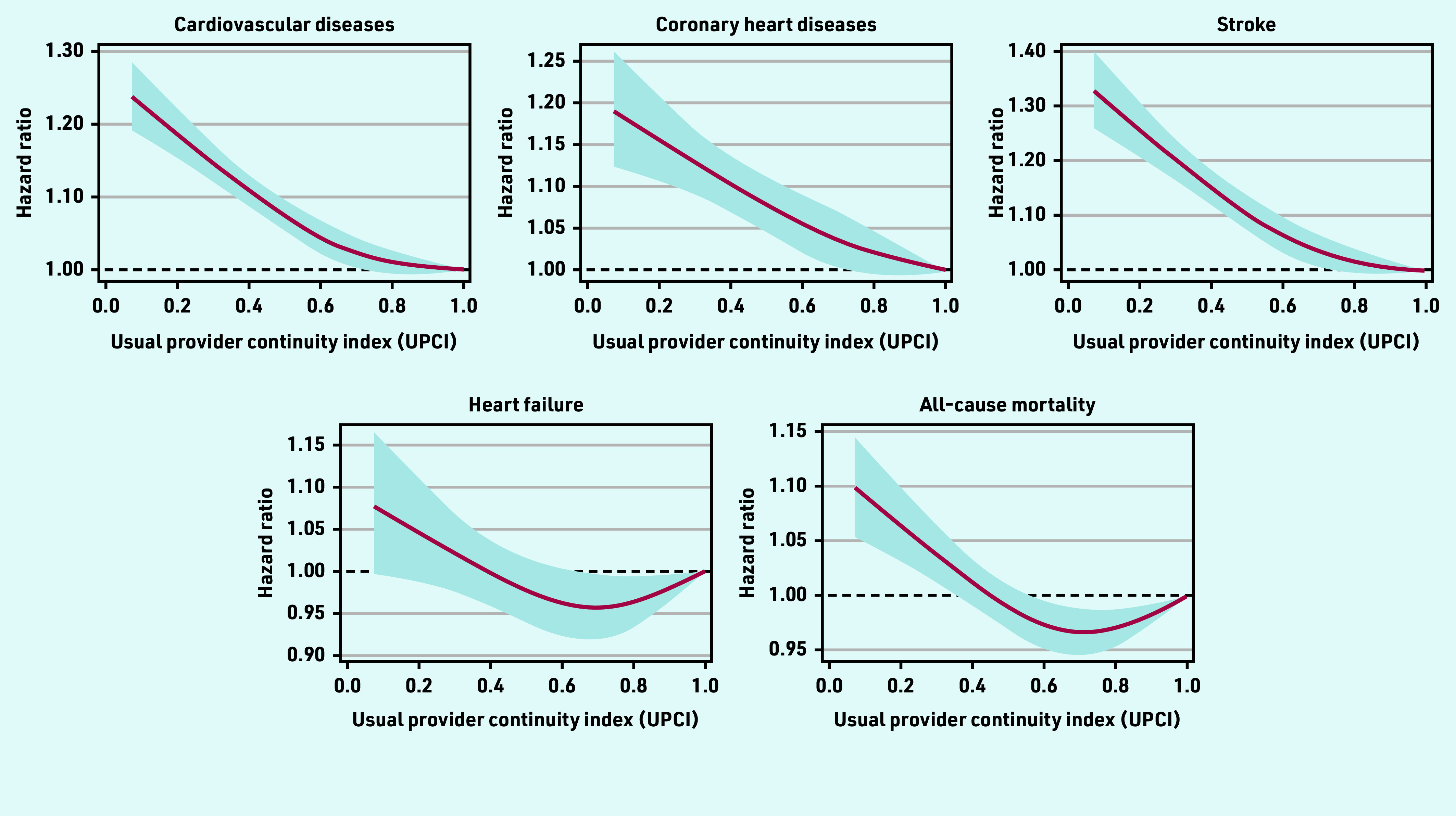
***Restricted cubic splines on the association between the usual provider of care index (UPCI) and the risks of cardiovascular diseases or mortality. Analysis was adjusted for age, sex, smoking status, systolic and diastolic blood pressure, body mass index, fasting glucose, low-density lipoprotein cholesterol, estimated glomerular filtration rate, Charlson comorbidity index, number of medical attendances, use of antihypertensive drugs, use of lipid-lowering drugs, and use of antidiabetic drugs at baseline***.

The HRs (95% confidence interval [CI]) for CVD for patients with UCPI in the second, third, and fourth quartile, relative to the reference group (UPCI<0.5), were 0.94 (95% CI = 0.92 to 0.96), 0.91 (95% CI = 0.89 to 0.93), and 0.90 (95% CI = 0.88 to 0.92) respectively (Figure 2). Similar patterns were observed for CHD and stroke. However, only a marginally statistically significant difference (0.95 [95% CI = 0.91 to 1.00]) was found for the risk of heart failure between the patients in the second quartile and the reference group. Risks of all-cause mortality were statistically slightly lower in patients with a moderate degree of COC (second and third quartile) compared with those in the reference group (HR: 0.96 [95% CI = 0.94 to 0.99] and 0.96 [95% CI = 0.93 to 0.98]), respectively. A linear negative association was observed between the UPCI and overall CVD risk, as well as the risk for CHD and stroke, but not for the heart failure and all-cause mortality, which suggested that the risk for heart failure and all-cause mortality did not decrease proportionally as the degree of team-based COC increased (Figure 3).

### Subgroup and sensitivity analysis

**Figure 4.**
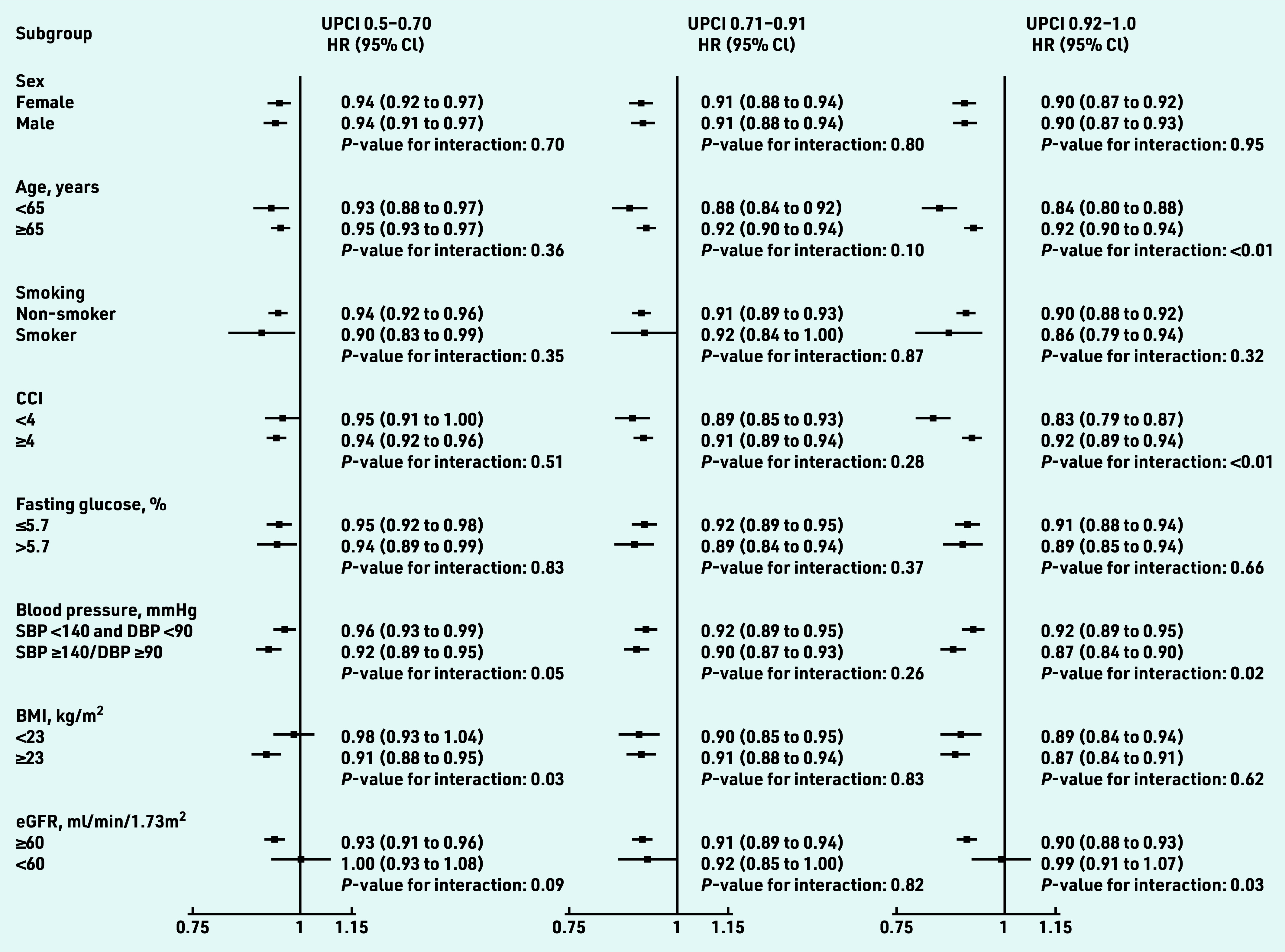
*Subgroup analyses on the association between team-based continuity of care and cardiovascular diseases among patients with hypertension. Hazard ratios were adjusted by age, sex, smoking status, SBP and DBP, BMI, fasting glucose, low-density lipoprotein cholesterol, eGFR, CCI, number of attendances, use of antihypertensive drugs, use of lipid-lowering drugs, and use of antidiabetic drug at baseline. CVD includes coronary heart disease, heart failure, and stroke. BMI = body mass index. CCI = Charlson comorbidity index. CVD = cardiovascular disease. DBP = diastolic blood pressure. eGFR = estimated glomerular filtration rate. HR = hazards ratio. SBP = systolic blood pressure. UPCI = usual provider of care index.*

Figure 4 illustrates the results from the subgroup analysis. The risk reduction of CVD among patients with higher team- based COC was preserved in most subgroups except for patients with baseline eGFR <60ml/min/1.73m^2^. For patients in the highest UPCI quartile, the association between the UPCI and the reduced risk of overall CVD was significantly stronger in patients with unsatisfactory blood pressure control at baseline (SBP ≥140 or DBP ≥90 mmHg *P *_interaction_ = 0.02), those aged <65 years ( *P *_interaction_ <0.01), and those with a CCI <4 ( *P *_interaction_ <0.01) in these subgroup analyses, and no significant differences in the effect size for COC was found between subgroups in terms of sex, smoking status, fasting glucose, and BMI ( *P *_interaction_>0.05). The risk of overall CVD was statistically significantly reduced in the groups in the higher quartiles of UPCI in all sensitivity analyses (Supplementary Figure S1).

## DISCUSSION

### Summary

Higher team-based COC was associated with reduced risk for overall CVD and all- cause mortality among patients with hypertension, especially for patients with poor blood pressure control at baseline. A linear association was observed between the degree of team-based COC and reduced risks of overall CVD, CHD, and stroke. A greater effect of team-based COC on CVD risk was observed in patients aged <65 years and those with fewer comorbidities (CCI <4). However, the protective effect of team-based COC was not as apparent for heart failure and all-cause mortality. Additionally, the prevention of CVD among patients with impaired kidney function was also less pronounced. These findings suggest that early initiation of team-based COC may provide additional advantages for patients with hypertension.

Team-based COC via a physician group can serve as a feasible and effective approach for achieving COC in healthcare systems with limited resources.

### Strengths and limitations

To the authors’ knowledge, this study is the first population-based cohort study to investigate the relationship between team- based COC and risks of CVD in patients with hypertension. The key strength of this study was the large sample size and long follow-up period, which enhanced the strength of the evidence on the long-term effects of team-based COC. Additionally, the authors included multiple endpoints and stratified patients with hypertension into subgroups, which provided a more detailed understanding of the effects of team-based COC among this highly heterogeneous population group.

There are several limitations in this study. First, clustering bias might exist since team-based COC was only implemented in some primary care clinics. However, standardised guidelines for hypertension management in primary care practices have been implemented in Hong Kong for over a decade,^24^ and thus differences in clinical practices across clinics should be negligible. Second, an intention-to-treat approach was adopted, in which possible changes in the degree of team-based COC during the follow-up period were accounted for in the analysis. The visit patterns were assumed to remain relatively stable since patients with chronic diseases generally have higher COC during follow-up care.^25^ Third, information on socioeconomic status was unavailable in the database used for confounding adjustment. However, the local public general outpatient clinics were heavily subsidised,^26^ so the bias in access to care should be limited. Additionally, the information to identify the individual-based COC (visit the same individual physician) was not available for analysis owing to a privacy policy, and thus the authors could not provide an examination of potential overlap between team-based COC and individual-based COC. However, the patients’ chance of visiting the same doctor during the long-term follow- up period and its influence on the analysis should be limited to a small extent because it was difficult to sustain the individual-based COC because of physicians’ temporary absences, rotations and retirement.

### Comparison with existing literature

The present study results are largely consistent with a previous cohort study investigating the effects of COC provided by the same medical clinics/hospitals in newly diagnosed patients with hypertension in Korea, where a higher COC index was associated with a reduced risk of overall CVD (adjusted HR: 0.76 [95% CI = 0.73 to 0.79]).^27^ Another cohort study in patients with newly diagnosed cardiovascular risk factors (including hypertension) also reported similar findings: participants with a COC index below the median had significantly greater risks of incident myocardial infarction (adjusted HR 1.57 [95% CI = 1.28 to 1.95]), ischemic stroke (1.44 [95% CI = 1.27 to 1.63]), as well as all-cause and cardiovascular mortality (adjusted HR: 1.12 [95% CI = 1.04 to 1.21] and 1.30 [95% CI = 1.13 to 1.50] respectively).^28^ Compared with these two studies, the authors have additionally observed a linear association between the UPCI and the risk reduction for overall CVD, CHD, and stroke, suggesting the potential benefits of intensifying the degree of team- based COC for patients with hypertension.

A stronger association between team- based COC and CVD risk reduction was found among patients with unsatisfactory blood pressure control, those aged <65 years, and those with fewer comorbidities (CCI <4) at baseline. It has been shown in the previous study that COC can help to achieve blood pressure control, and thus improve the overall health of patients with hypertension.^6^ As an alternative pattern for individual-based COC, the team-based COC can be more effective for patients with poor blood pressure control, who deserve higher priority in the delivery of COC service. The cohort study conducted by Choi *et al* similarly reported a slightly larger effect size of higher COC on CVD prevention among patients aged <60 years (adjusted HR: 0.72 [95% CI = 0.68 to 0.76] compared with those aged ≥60 years (adjusted HR: 0.77 [95% CI = 0.74 to 0.80]).^27^ Older patients and those with more comorbidities were more likely to have deteriorated health status, for whom the team-based COC might have limited efficacy on the endpoint of CVD incidence. However, the risk reduction for CVDs in these patients was still significant and noteworthy given their population size and the higher incidence rates for CVDs. Further studies are also needed to investigate other efficacy endpoints, such as the improvement in quality of life, for a more comprehensive understanding of the benefits of team-based COC for patients with different health status. From another perspective, the relatively stronger effect of team-based COC in younger patients and those with fewer comorbidities suggested that early initiation of team-based COC during a patient’s course of treatment, or before the deterioration of health status, would contribute to greater benefits in preventing future CVD events, which follows the principle of early prevention for CVD.^29^ Additional attention should be directed towards younger adult patients to advocate for a continuous relationship with their physician team, in order to maximise the benefits of team-based COC.

The insignificant effect of team-based COC among patients with hypertension with impaired kidney function could be due to their deteriorated health status, thus higher underlying risk for CVD.^30^ A previous study on patients with hypertension and chronic kidney disease showed that greater COC from the same GP could increase the likelihood of achieving the recommended blood pressure targets.^5^ Thus, further studies are still needed to evaluate the effectiveness of team-based COC in this patient subgroup since hypertension frequently coexisted with CKD.^31^ Additionally, the protective effect of team-based COC for heart failure and all-cause mortality was insignificant in the highest UPCI quartile. Patients with hypertension and a higher risk of heart failure and/or all-cause mortality were more likely to have deteriorated health and tended to seek care from the same provider, such as the patients at the end-of-life stage,^32^ for whom further intensification in team-based COC might have been of limited efficacy on reducing the risks of relatively severe endpoints such as heart failure and all-cause mortality.

### Implications for practice

This study showed clinically significant effects of team-based COC in reducing the risks of CVD incidence and mortality among patients with hypertension. Patients with hypertension require life- long follow- up visits to monitor the treatment and refill medications.^33^ Team- based COC can transform the previous one- to- one physician–patient relationship by connecting the patients to a coordinated group of physicians, thereby helping to sustain COC for patients with hypertension in real-life clinical practice. By working collaboratively with other team members, physicians can ensure the long- term continuity in disease monitoring (for example, blood pressure and medication effect) and patients’ adherence to the treatment plan, which can ultimately benefit the patients’ health outcomes in the long run. Additionally, team-based COC can also improve communications among physicians, allowing them to share their knowledge and expertise related to the treatment of the patients, which can help the physicians develop a shared understanding of the best practices for managing specific patients and ultimately benefit patient outcomes.

Theoretically, the benefits of team- based COC should be transferable across common chronic conditions in primary care. COC is critical in successful chronic disease management and the COC provided by a coordinated physician team enhances the sustainability of COC for the conditions that required long-term follow- up, such as type 2 diabetes mellitus,^14^ hypertension, chronic obstructive pulmonary disease, and so on. Related policies will be needed to support the team-based COC in individualised health systems. The development of coordinated physician teams and workforce training are crucial components in human resource management to realise team-based COC. The enhancement of the infrastructure and the supporting system is also critical in improving the efficiency of team-based COC delivery in both informational and managerial aspects, which includes upgrades to information technology to improve information sharing within the physician team and facilitate the appointment schedule for patients seeking care from the same physician teams.^13^

## Data

The data underlying this article were provided by the Hospital Authority (HA) of Hong Kong and can be accessed upon request to the HA of Hong Kong (https://www3.ha.org.hk/data/Provision/ApplicationProcedure).
